# Supplementary material for: Structural basis for MTA1c-mediated DNA N6-adenine methylation
Source: Nat Commun. 2022 Jun 7;13:3257. doi: 10.1038/s41467-022-31060-6 (PMC9174199; doi:10.1038/s41467-022-31060-6)
Supplement: Supplementary file 1 — Supplementary Information [file 41467_2022_31060_MOESM1_ESM.pdf]

Supplementary Figures

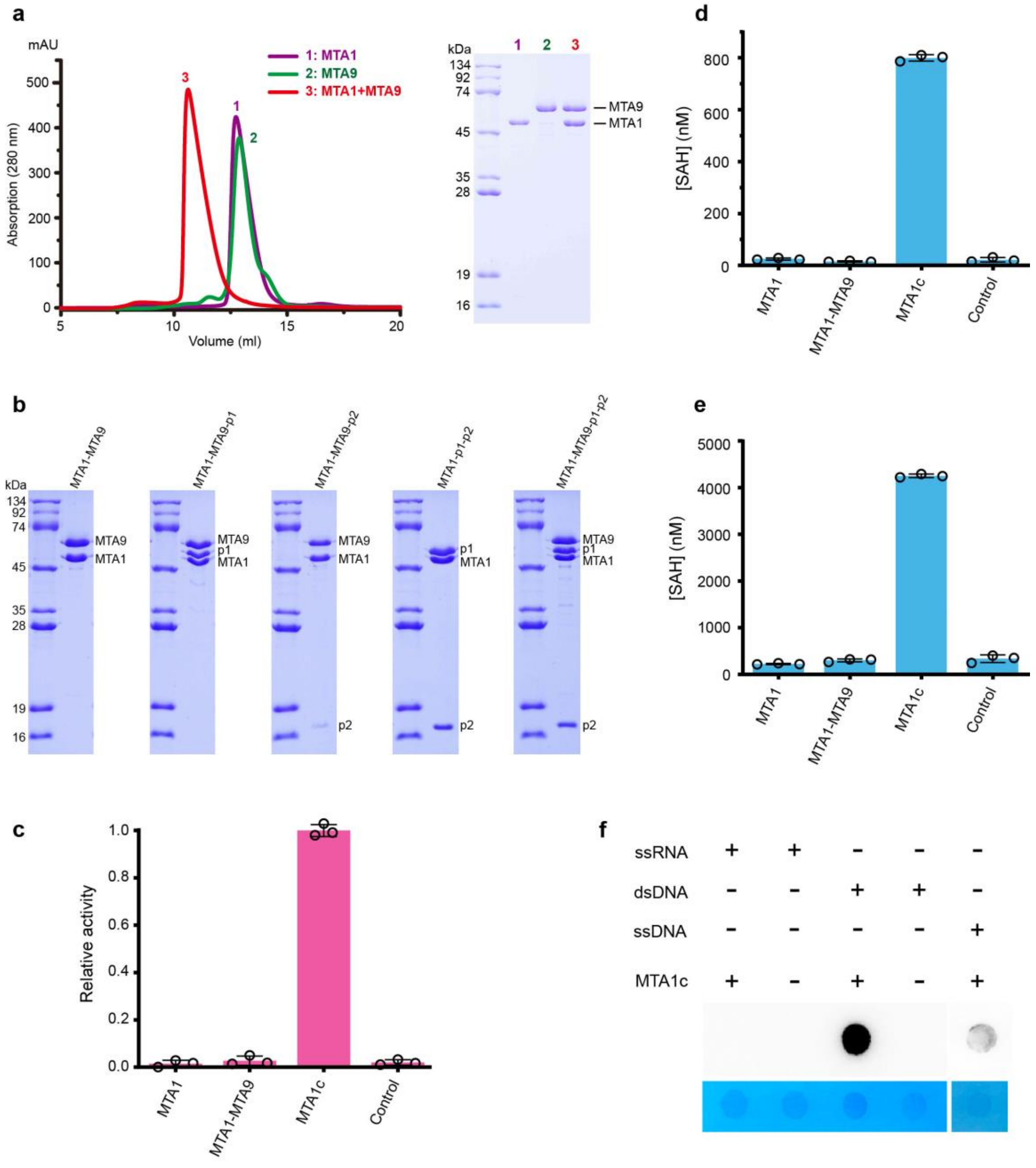

Supplementary Figure 1

Supplementary Fig. 1 Reconstitution of TthMTA1c complex and its' subcomplexes. a Gel filtration chromatography shows that TthMTA1 interacts with TthMTA9. All samples were fractionated on a Superdex 200 increase

10/300, and the peak fractions were analyzed by SDS-PAGE. **b** Purification of four-component TthMTA1c complex, as well as two- and three-component subcomplexes. All complexes were expressed in *Escherichia coli* and copurified by Ni^{2+}-agarose affinity column chromatography followed by size exclusion chromatography. **c** Antibody-based methyltransferase activity assay using oligonucleotide. Data are shown as mean ± SD from n = 3 independent experiments; open circles indicate values for individual repeat measurements. Control, in the absence of MTA1c. **d** and **e** SAM-dependent methyltransferase assay (MTase-Glo assay) using 59-bp oligonucleotide (**d**) and 954-bp dsDNA (**e**). The 954-bp dsDNA was amplified by PCR from *Tetrahymena thermophila* strain SB210 genomic DNA. Data are shown as mean ± SD from n = 3 independent experiments; open circles indicate values for individual repeat measurements. **f** Antibody-based methyltransferase assay using dsDNA, ssDNA, and RNA substrates. Source data are provided as a Source Data file.

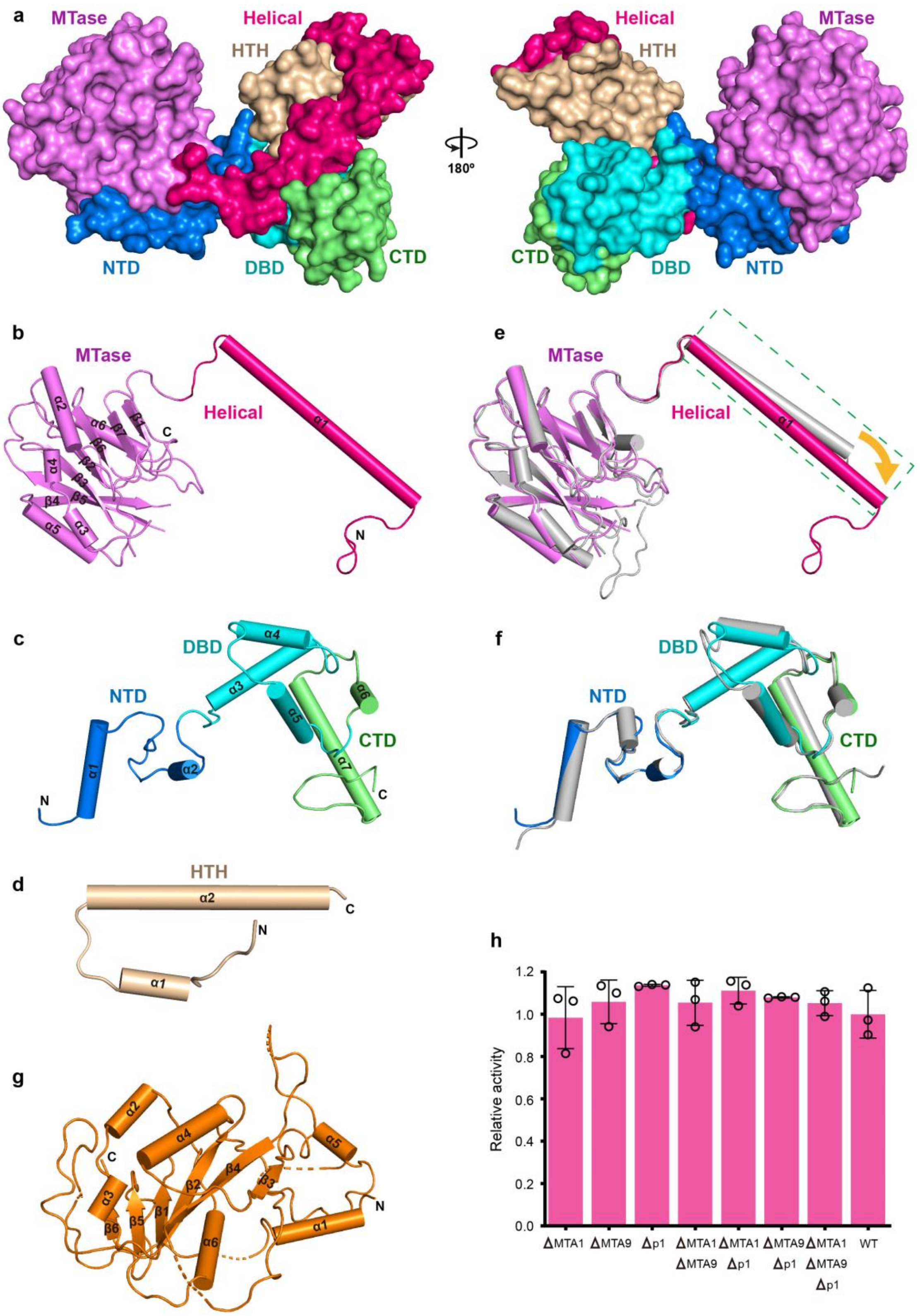

Supplementary Figure 2

Supplementary Fig. 2 Architecture of TthMTA1-Tthp1-Tthp2, TthMTA1-Tthp2 and TthMTA1-PteMTA9 complexes. a Front and rear views of the

overall structure of the MTA1-p1-p2 ternary complex in surface representation. **b-d** Overall structures of MTA1 subunit (**b**), p2 subunit (**c**) and p1 subunit (**d**). **e** Structural comparison of the MTA1 subunit in the MTA1-p1-p2 ternary complex and the MTA1 subunit (gray) in the MTA1-p2 binary complex. **f** Structural comparison of the p2 subunit in the MTA1-p1-p2 ternary complex and the p2 subunit (gray) in the MTA1-p2 binary complex. **g** Overall structure of PteMTA9 subunit. **h** *In vitro* methyltransferase assay using indicated truncations. A 954-bp dsDNA PCR product was used as substrate in the assay. Antibody-based methyltransferase activity assay was used for the detection. The proteins used in the experiment were all from *Tetrahymena thermophila*. "Δ" indicated the MTA1c protein was truncated, specifically as follows: ΔTthMTA1 (residues 126-372), ΔTthMTA9 (residues 67-449) and ΔTthp1 (residues 1-309). Data are shown as mean ± SD from n = 3 independent experiments; open circles indicate values for individual repeat measurements. Source data are provided as a Source Data file.

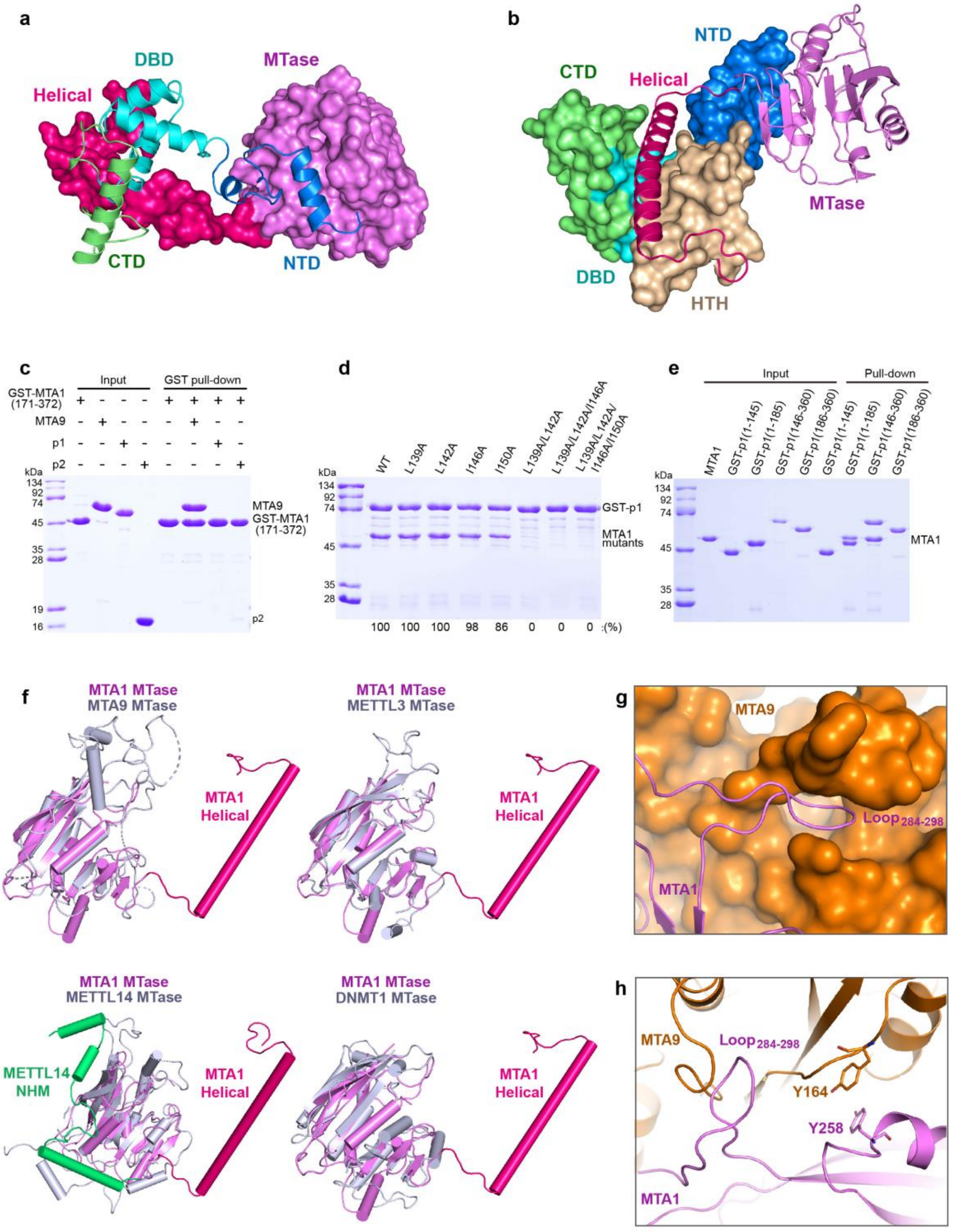

Supplementary Figure 3

Supplementary Fig. 3 The Helical domain of TthMTA1 interacts with Tthp1 and Tthp2. a p2 binding in the cavity formed between the MTase and Helical domains of MTA1. **b** The Helical domain of MTA1 binding in the groove formed between the CTD domain of p2 and the HTH domain of p1. **c** GST pull-down

experiments assaying the ability of the MTase domain of MTA1 to interact with MTA9, p1 and p2. **d** GST pull-down experiments assaying the ability of MTA1 mutants to interact with GST-p1. **e** GST pull-down experiments assaying the ability of N-terminal or C-terminal truncated p1 proteins (GST tag) to interact with MTA1. **f** Structural comparison of the MTase domains of MTA1 and MTA9 (top left panel), MTA1 and METTL3 (top right panel), MTA1 and METTL14 (bottom left panel), MTA1 and DNMT1 (bottom right panel). The MTase domain and the Helical domain of MTA1 are shown in violet and hotpink, respectively. The MTase domains of MTA9, METTL3, METTL14 and DNMT1 are shown in gray. The N-terminal α-helical motif (NHM) is shown in green. **g** The loop (residues 284-298) of MTA1 is stabilized by MTA9. **h** The side chain of Tyr164 within MTA9 forms stacking interaction with the side chain of Tyr258 within MTA1. Source data are provided as a Source Data file.

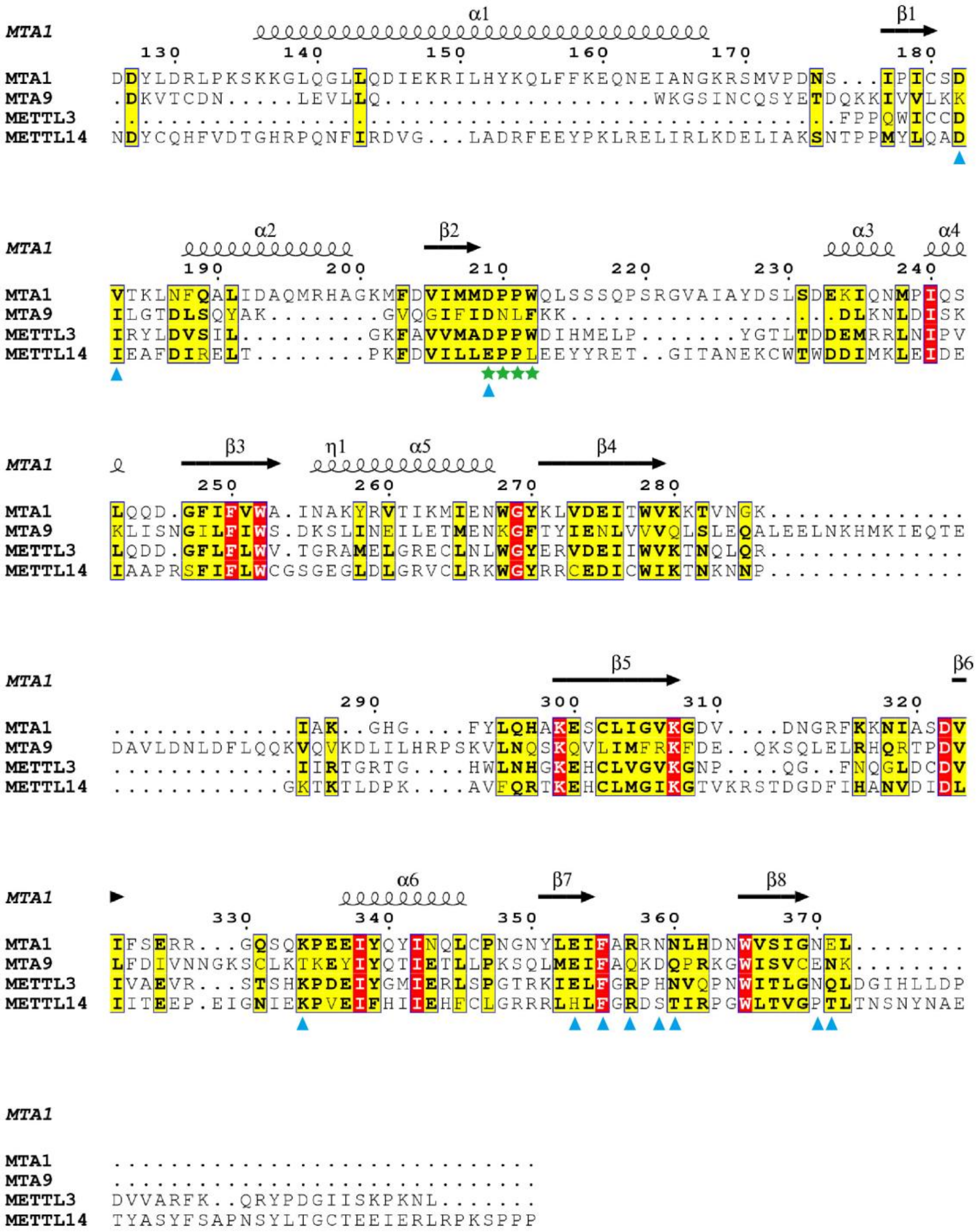

Supplementary Figure 4

Supplementary Fig. 4 Sequence alignment of TthMTA1, PteMTA9, human METTL3 and METTL14. Alignment was performed using the MUSCLE program. The secondary structure of MTA1 is shown on top. Conserved residues are shaded in yellow, and invariant residues are shown in red. Residues involved in SAM binding for MTA1 are marked in blue triangles. The catalytic residues are highlighted by green stars.

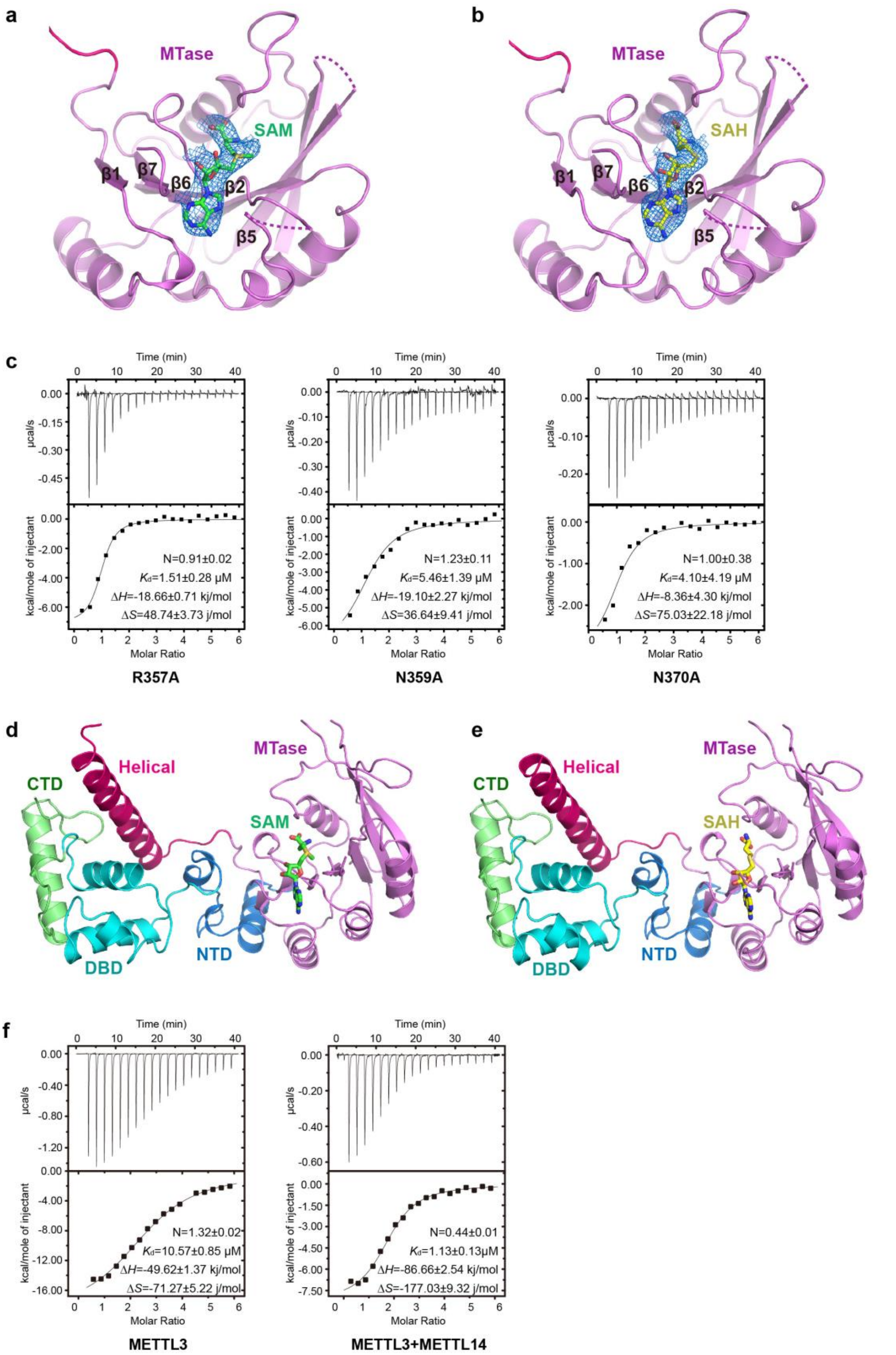

Supplementary Figure 5

Supplementary Fig. 5 Recognition of the SAM and SAH. a Electron density

map of the SAM. The 2mFo-Fc electron density map of the SAM is shown as a blue mesh (contoured at 1.0 σ). The SAM is shown in stick representation. The MTase domain of TthMTA1 is shown in cartoon representation. **b** Electron density map of the SAH. The 2mFo-Fc electron density map of the SAH is shown as a blue mesh (contoured at 1.0 σ). The SAH is shown in stick representation. The MTase domain of TthMTA1 is shown in cartoon representation. **c** ITC assay of the TthMTA1c complexes with indicated MTA1 point mutations. Each MTA1-MTA9-p1-p2 mutant complex used for ITC assay was purified as MTA1-MTA9-p1-p2 WT complex. **d** Ribbon representation of TthMTA1-Tthp2 in complex with SAM. **e** Ribbon representation of TthMTA1-Tthp2 in complex with SAH. **f** ITC assay with human METTL3 and METTL3-METTL14 complex.

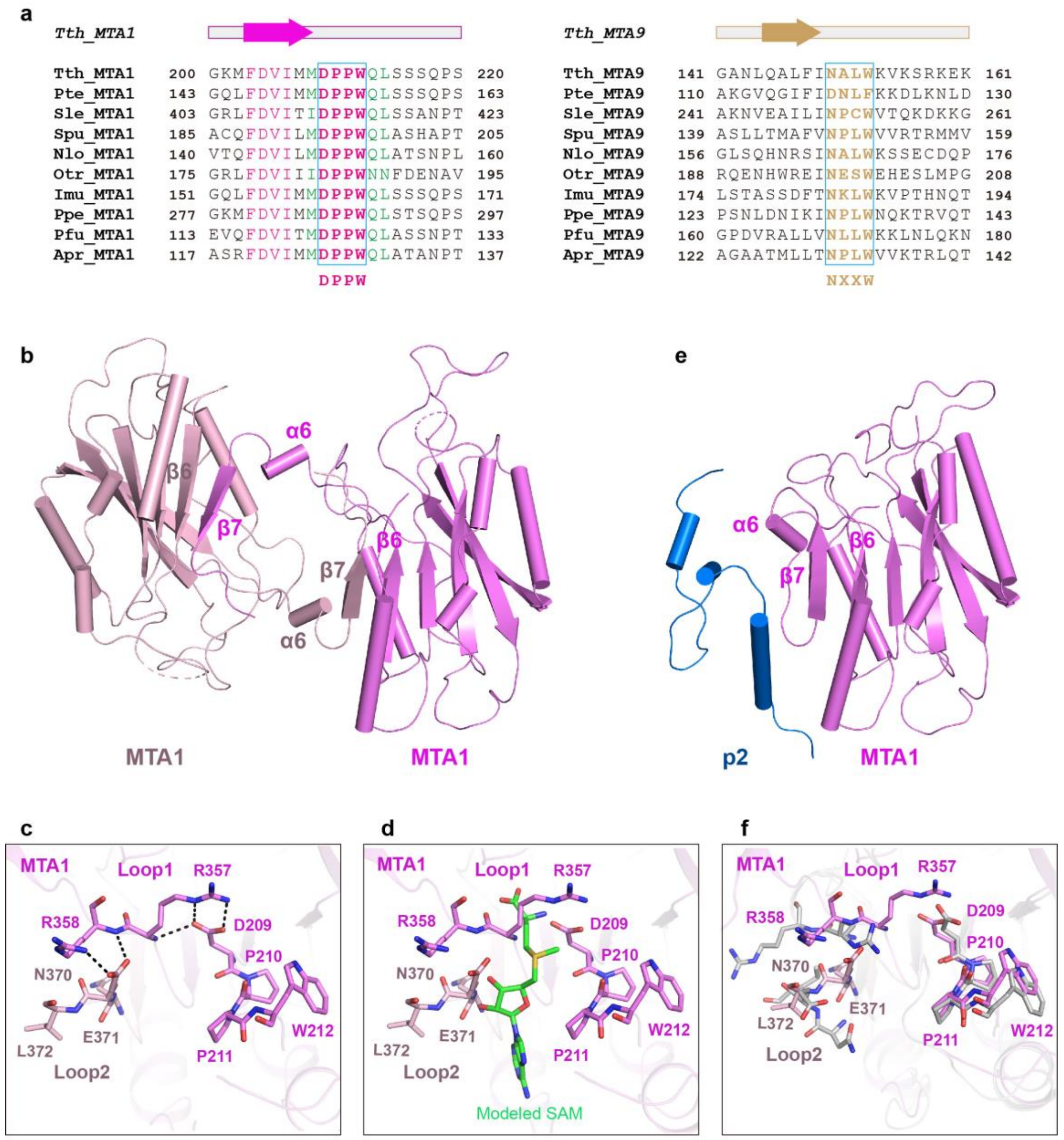

Supplementary Figure 6

Supplementary Fig. 6 Conformational change in the SAM binding pocket of MTA1. a Sequence alignments of catalytic motifs in the MTA1 (left panel) and MTA9 (right panel) proteins. **b** The overall structure of the MTA1 MTase domain in ribbon representation. In the crystal, the β7 strand from one MTA1 molecule (violet) form a β-sheet with the β1-β6 strands of the neighboring MTA1 molecule (lightpink). **c** The SAM-binding pocket is formed by two neighboring MTA1 molecules in MTA1 structure. **d** The Loop1 and Loop2 within the SAM-binding pocket of MTA1 show clashes with the modeled SAM. **e** The β7 strand

of MTA1 is stabilized by the NTD domain of p2 in the structure of MTA1-p2 complex. **f** Structural comparison of the SAM-binding pocket of MTA1 in the p2-free state (violet) and the p2-bound state (gray).

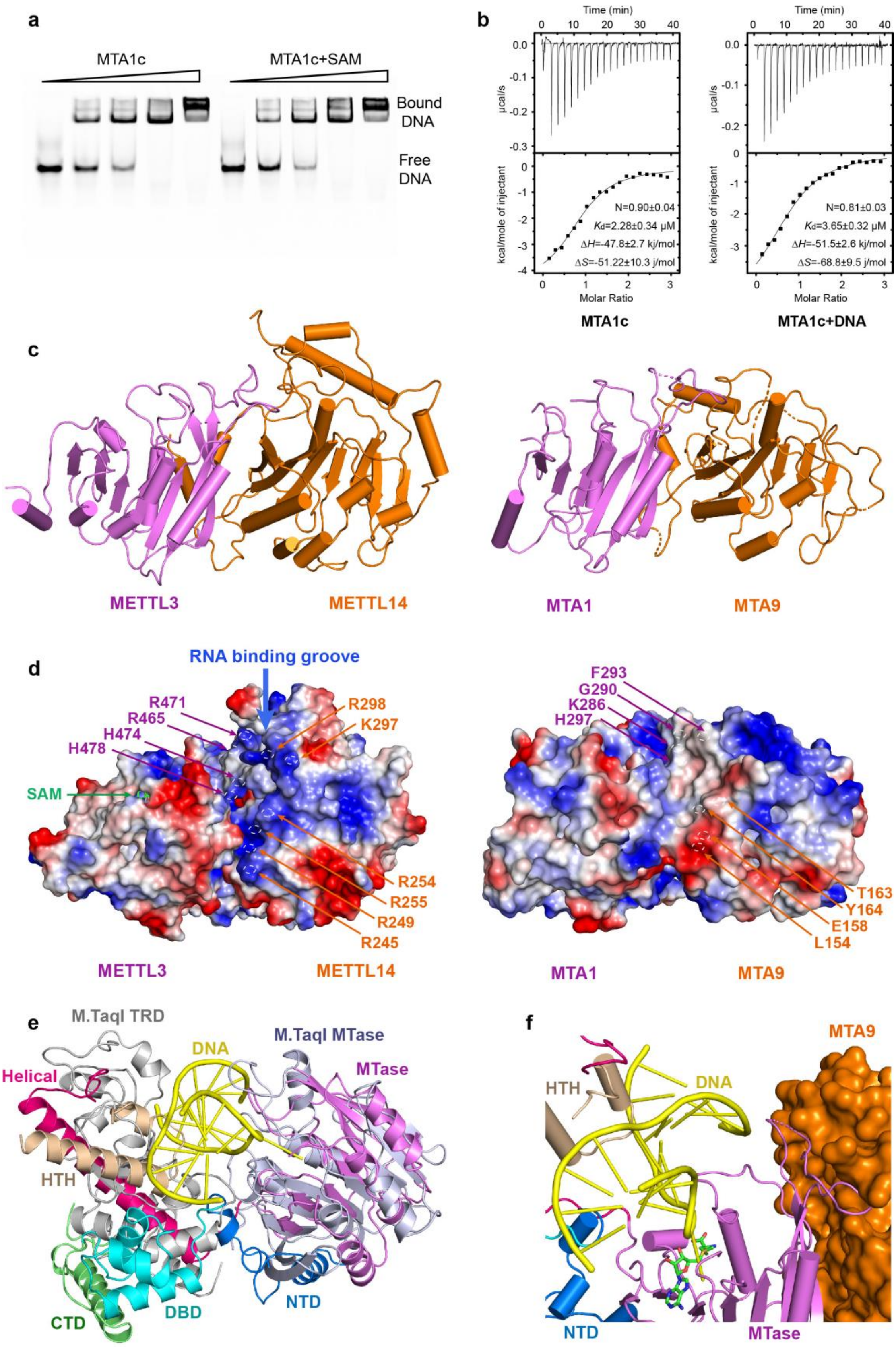

Supplementary Figure 7

Supplementary Fig. 7 MTA1c has distinct DNA binding mode. a Effects of

SAM binding on DNA affinity. **b** Effects of DNA binding on SAM affinity. **c** Comparison of the overall structures of METTL3-METTL14 (left panel) and MTA1-MTA9 (right panel) complexes. **d** The surface electrostatic potentials of METTL3-METTL14 (left panel) and MTA1-MTA9 (right panel) complexes. **e** Superimposition of the M.TaqI-DNA complex structure (PDB: 1G38) onto the MTA1-p1-p2 complex. **f** MTA9 stabilizes the putative DNA binding channel of MTA1c. Source data are provided as a Source Data file.

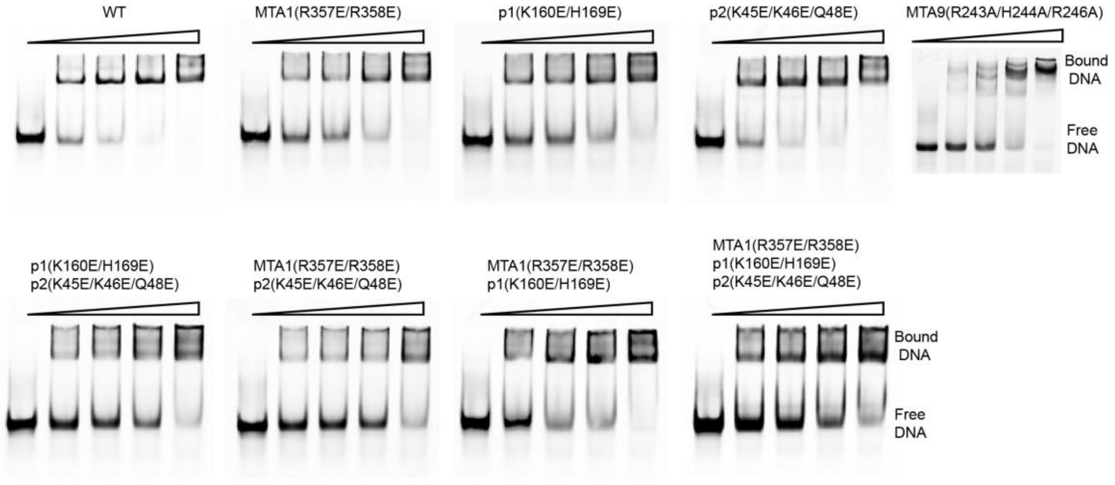

Supplementary Figure 8

Supplementary Fig. 8 Electrophoretic mobility shift assay of the MTA1-MTA9-p1-p2 complexes with indicated MTA1, MTA9, p1 or p2 mutations.

Source data are provided as a Source Data file.

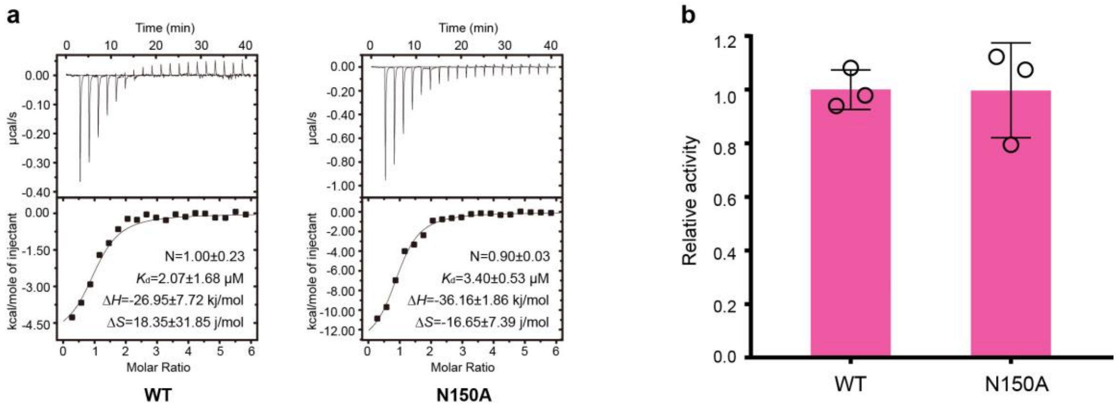

Supplementary Figure 9

Supplementary Fig. 9 Effects of Asn150 substitution of MTA9 in TthMTA1c complex on SAM binding (a) and DNA methylation (b). Asn150 is the potential catalytic residue of TthMTA9. For DNA methylation assay, data are shown as mean ± SD from n = 3 independent experiments; open circles indicate values for individual repeat measurements. Source data are provided as a Source Data file.

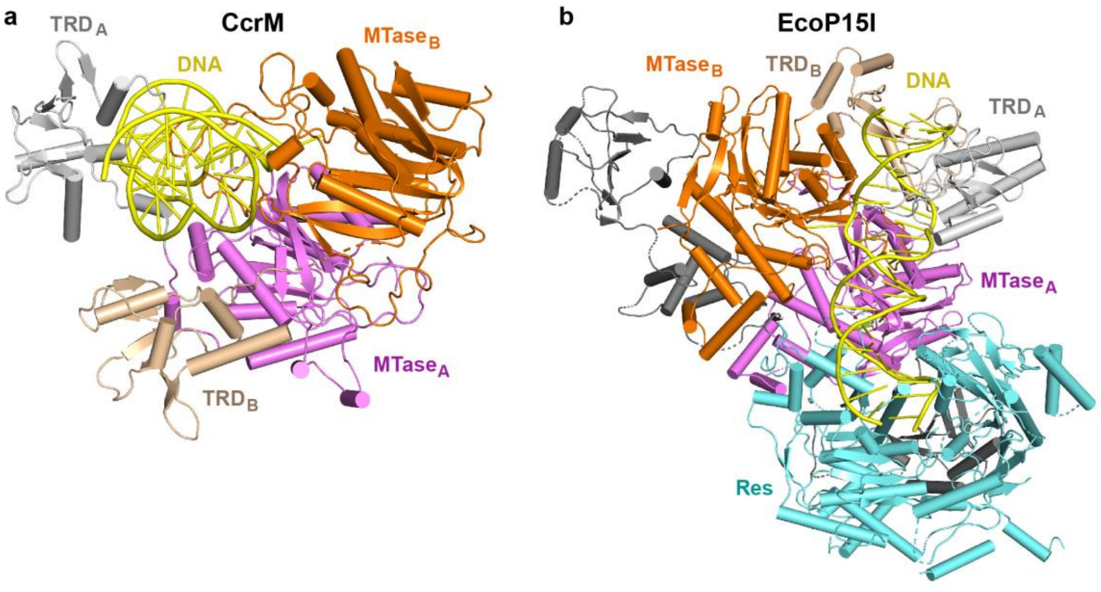

Supplementary Figure 10

Supplementary Fig. 10 The TRD domain is required for CcrM (a) and Ecop15I (b) to bind DNA substrate.

Supplementary Tables

Supplementary Table 1. Crystallographic data collection and refinement statistics.

	Se_MTA1-p1-p2	MTA1-p1-p2-SAM	MTA1-p1-p2-SAH	MTA1-PteMTA9
**Data collection***				
Space group	$P2_12_12_1$	$P2_12_12_1$	$P2_12_12_1$	$P2_1$
Cell dimensions				
a, *b*, *c* (Å)	82.8, 86.5, 161.0	83.5, 85.8, 162.9	82.6, 84.9, 160.1	75.7, 75.6, 142.5
α, β, γ (°)	90.0, 90.0, 90.0	90.0, 90.0, 90.0	90.0, 90.0, 90.0	90.0, 99.2, 90.0
Resolution (Å)	50.00-2.72 (2.77-2.72)	50.00-3.40 (3.46-3.40)	50.00-3.00 (3.05-3.00)	50.00-3.10 (3.15-3.10)
R_{merge}	0.135 (0.989)	0.107 (0.968)	0.213 (0.953)	0.337 (0.992)
$I/\sigma I$	31.0 (3.2)	20.3 (1.9)	6.2 (1.0)	5.2 (1.3)
Completeness (%)	100 (100)	100 (100)	99.0 (98.9)	99.3 (99.2)
Redundancy	21.6 (17.7)	10.9 (9.1)	5.0 (4.0)	5.4 (4.6)
**Refinement**				
Resolution (Å)	2.72	3.58	3.12	3.10
No. reflections	31,233	13,242	14,829	32,542
R_{work} / R_{free}	0.2399/0.2538	0.2507/0.2758	0.2424/0.2717	0.3037/0.3156
No. atoms				
protein	6,084	6,115	6,115	8,934
Ligand	0	54	52	0
Water	58	24	35	212
B-factors (Å^2)				
Protein	32.0	61.8	46.4	65.80
Ligand	0	60.8	38.5	0
Water	29.1	18.2	27.2	48.79
R.m.s. deviations				
Bond length (Å)	0.018	0.013	0.012	0.010
Bond angles (°)	1.771	1.360	1.360	1.654
Ramachandran plot				
Favored region	97.81	96.86	96.72	94.71
Allowed region	2.19	3.14	3.28	5.29
Outlier region	0.00	0.00	0.00	0.00

* Highest resolution shell is shown in parentheses.

Supplementary Table 2. Crystallographic data collection and refinement statistics.

	Se_MTA1-p2	MTA1-p2-SAM	MTA1-p2-SAH
**Data collection***			
Space group	$P3_221$	$P3_221$	$P3_221$
Cell dimensions			
a, b, c (Å)	137.4, 137.4, 61.6	137.0, 137.0, 61.6	136.8, 136.8, 60.9
α, β, γ (°)	90.0, 90.0, 120.0	90.0, 90.0, 120.0	90.0, 90.0, 120.0
Resolution (Å)	50.00-3.10 (3.15-3.10)	50.00-2.97 (3.02-2.97)	50.00-3.40 (3.46-3.40)
R_{merge}	0.136 (0.988)	0.084 (0.950)	0.229 (0.896)
$I/\sigma I$	37.5 (4.0)	28.8 (2.2)	11.0 (1.5)
Completeness (%)	100 (100)	100 (100)	99.8 (99.5)
Redundancy	33.2 (30.1)	13.0 (11.5)	6.9 (5.7)
**Refinement**			
Resolution (Å)	3.10	2.98	3.42
No. reflections	12,112	12,001	7,170
R_{work} / R_{free}	0.2491/0.2739	0.2488/0.2649	0.2491/0.2628
No. atoms			
protein	2,769	2,776	2,756
Ligand	0	27	26
Water	17	18	15
B-factors (Å^2)			
Protein	49.9	39.7	30.2
Ligand	0	34.2	23.2
Water	42.6	22.8	11.6
R.m.s. deviations			
Bond length (Å)	0.016	0.017	0.016
Bond angles (°)	1.645	1.595	1.683
Ramachandran plot			
Favored region	97.93	97.64	97.92
Allowed region	2.07	2.36	2.08
Outlier region	0.00	0.00	0.00

* Highest resolution shell is shown in parentheses.

Supplementary Table 3. Crystallographic data collection and refinement statistics.

	MTA1-Ptep2-SAM	MTA1 (171-372)
**Data collection***		
Space group	$P2_1$	$P2_1$
Cell dimensions		
a, *b*, *c* (Å)	98.1, 110.2, 102.6	44.9, 112.9, 81.9
α, β, γ (°)	90.0, 91.6, 90.0	90.0, 97.8, 90.0
Resolution (Å)	50.00-3.65 (3.71-3.65)	50.00-1.83 (1.86-1.83)
R_{merge}	0.123 (0.935)	0.113 (0.755)
$I/\sigma I$	10.3 (1.1)	11.8 (1.4)
Completeness (%)	99.4 (98.5)	92.5 (92.4)
Redundancy	4.6 (3.9)	5.1 (4.0)
**Refinement**		
Resolution (Å)	3.68	1.83
No. reflections	18,100	54,422
R_{work} / R_{free}	0.2821/0.3284	0.2099/0.2406
No. atoms		
protein	10,605	5,719
Ligand	108	0
Water	12	541
B-factors (Å^2)		
Protein	61.6	25.6
Ligand	36.8	0
Water	32.7	31.0
R.m.s. deviations		
Bond length (Å)	0.008	0.014
Bond angles (°)	1.016	1.404
Ramachandran plot		
Favored region	93.87	98.10
Allowed region	6.13	1.90
Outlier region	0.00	0.00

* Highest resolution shell is shown in parentheses.
