# Supplementary material for: Structural basis for MTA1c-mediated DNA N6-adenine methylation
Source: Nat Commun. 2022 Jun 7;13:3257. doi: 10.1038/s41467-022-31060-6 (PMC9174199; doi:10.1038/s41467-022-31060-6)
Supplement: Supplementary file 3 — Reporting Summary [file 41467_2022_31060_MOESM3_ESM.pdf]

nature portfolio

Corresponding author(s): Liang Liu

Last updated by author(s): May 9, 2022

Reporting Summary

Nature Portfolio wishes to improve the reproducibility of the work that we publish. This form provides structure for consistency and transparency in reporting. For further information on Nature Portfolio policies, see our Editorial Policies and the Editorial Policy Checklist.

Statistics

For all statistical analyses, confirm that the following items are present in the figure legend, table legend, main text, or Methods section.

n/a	Confirmed	
☐	☒	The exact sample size (*n*) for each experimental group/condition, given as a discrete number and unit of measurement
☐	☒	A statement on whether measurements were taken from distinct samples or whether the same sample was measured repeatedly
☒	☐	The statistical test(s) used AND whether they are one- or two-sided<br>*Only common tests should be described solely by name; describe more complex techniques in the Methods section.*
☒	☐	A description of all covariates tested
☒	☐	A description of any assumptions or corrections, such as tests of normality and adjustment for multiple comparisons
☐	☒	A full description of the statistical parameters including central tendency (e.g. means) or other basic estimates (e.g. regression coefficient) AND variation (e.g. standard deviation) or associated estimates of uncertainty (e.g. confidence intervals)
☒	☐	For null hypothesis testing, the test statistic (e.g. *F*, *t*, *r*) with confidence intervals, effect sizes, degrees of freedom and *P* value noted<br>*Give P values as exact values whenever suitable.*
☒	☐	For Bayesian analysis, information on the choice of priors and Markov chain Monte Carlo settings
☒	☐	For hierarchical and complex designs, identification of the appropriate level for tests and full reporting of outcomes
☒	☐	Estimates of effect sizes (e.g. Cohen's *d*, Pearson's *r*), indicating how they were calculated

Our web collection on statistics for biologists contains articles on many of the points above.

Software and code

Policy information about availability of computer code

Data collection	The X-ray diffraction data sets were collected at beamline BL-17U1, BL-18U1 and BL-19U1 at Shanghai Synchrotron Radiation Facility (SSRF) in China.
Data analysis	The diffraction datasets were processed with HKL3000 (v720) package.<br>PHENIX (1.17.1-3660) AutoSol was used to generate an initial model.<br>PHENIX (1.17.1-3660) PHASER was used for molecular replacement.<br>PHENIX (1.17.1-3660) Refine was used for refinement.<br>WinCoot (0.7.2.1) was used for manual building of atomic models.<br>SWISS-MODEL server was used to generate a homology model.<br>PyMOL (2.4.0) was used for graphics.<br>Origin Pro 9.1 was used for statistical analyses.

For manuscripts utilizing custom algorithms or software that are central to the research but not yet described in published literature, software must be made available to editors and reviewers. We strongly encourage code deposition in a community repository (e.g. GitHub). See the Nature Portfolio guidelines for submitting code & software for further information.

Data

Policy information about availability of data

All manuscripts must include a data availability statement. This statement should provide the following information, where applicable:

- Accession codes, unique identifiers, or web links for publicly available datasets
- A description of any restrictions on data availability
- For clinical datasets or third party data, please ensure that the statement adheres to our policy

The atomic coordinates included in this study have been deposited in the Protein Data Bank (PDB) with the following accession codes: 7F4L, 7F4M, 7F4N, 7F4O, 7F4P, 7F4Q, 7F4R, 7F4S, and 7F4T.

Field-specific reporting

Please select the one below that is the best fit for your research. If you are not sure, read the appropriate sections before making your selection.

☒ Life sciences ☐ Behavioural & social sciences ☐ Ecological, evolutionary & environmental sciences

For a reference copy of the document with all sections, see nature.com/documents/nr-reporting-summary-flat.pdf

Life sciences study design

All studies must disclose on these points even when the disclosure is negative.

Sample size

For antibody-based methyltransferase assays (Figures 1c, 4c, 7b, 7f, Supplementary Figures 1c, 1f, 2h, 9b):
assay volume, 50 µl, pH 7.5, NaCl concentration 200 mM, EDTA concentration 6 mM, SAM concentration 160 µM, substrate dsDNA or RNA concentration 2.7 µg, reaction time 3 h, reaction temperature 37 °C.
For SAM-dependent methyltransferase activity assays (Supplementary Figures 1d, 1e):
assay volume, 50 µl, pH 7.5, NaCl concentration 200 mM, EDTA concentration 6 mM, SAM concentration 160 µM, 954-bp dsDNA concentration 2.7 µg, 59-bp oligo dsDNA 50 uM, reaction time 4 h, reaction temperature 37 °C.
For GST pull-down (Figures 1a, 3b-3d, 3g, 3h, Supplementary Figures 3c-3e):
GST-tagged protein 0.2 mg, untagged protein 0.4 mg, pH 7.5, NaCl concentration 300 mM, DTT concentration 2 mM, temperature 4 °C, time 60 min.
For size-exclusion chromatography assays (Figure 1b, Supplementary Figure 1a):
pH 7.5, NaCl concentration 300 mM, DTT concentration 2 mM, sample amount 1-2 mg.
For isothermal titration calorimetry assays (Figures 4d, 5a, 5c, 5d, Supplementary Figures 5c, 5f, 7b, 9a):
temperature 25 °C, protein concentration 20-40 µM, SAM concentration 400-1200 µM, pH 7.5, NaCl concentration 300 mM.
For electrophoretic mobility shift assays (Figure 7a, Supplementary Figures 7a, 8):
dsDNA concentration 4 µM, protein concentration 0-40 µM, pH 7.5, NaCl concentration 300 mM, temperature 4 °C, time 30 min.

Data exclusions

None

Replication

Antibody-based methyltransferase activity assays, SAM-dependent methyltransferase activity assays, GST pull-down, size-exclusion chromatography assays and electrophoretic mobility shift assays were repeated at least three times ($n \geq 3$) with similar results. Isothermal titration calorimetry assays were carried out at least twice ($n \geq 2$) with similar results.

Randomization

Structure refinements were performed with 5% randomly chosen reflections for validation by R-free values.
For methyltransferase activity assays, GST pull-down, size-exclusion chromatography assays, ITC and EMSA, no randomization was necessary as only single variable changed per experiment.

Blinding

X-ray diffraction data were measured quantitatively. Positive and negative controls were included in each biochemistry experiment for single variable parameter. Thus, we were not blind to these data.

Reporting for specific materials, systems and methods

We require information from authors about some types of materials, experimental systems and methods used in many studies. Here, indicate whether each material, system or method listed is relevant to your study. If you are not sure if a list item applies to your research, read the appropriate section before selecting a response.

Materials & experimental systems

n/a	Involved in the study	
☐	☒	Antibodies
☐	☒	Eukaryotic cell lines
☒	☐	Palaeontology and archaeology
☒	☐	Animals and other organisms
☒	☐	Human research participants
☒	☐	Clinical data
☒	☐	Dual use research of concern

Methods

n/a	Involved in the study	
☒	☐	ChIP-seq
☒	☐	Flow cytometry
☒	☐	MRI-based neuroimaging

Antibodies

Antibodies used

Anti-N6-methyladenosine antibody (Synaptic Systems, Cat #: 202003)
Goat Anti-rabbit IgG/HRP (Bioss, Cat #: bs-0295G-HRP)

Validation

Methyltransferase activity assays dot blot using rabbit anti-N6-methyladenosine polyclonal antibody for DNA and RNA samples has been validated by the manufacturer.

Eukaryotic cell lines

Policy information about cell lines

Cell line source(s)

Tetrahymena thermophila strain SB210 (the gift from the lab of professor Wenjing Zhang (College of Ocean and Earth Sciences, Xiamen University, China)).
Spodoptera frugiperda Sf9 cells (the gift from the lab of professor Caihong Yun (Department of Biochemistry and Biophysics, Peking University Health Science Center, China)).

Authentication

None of the cell lines used were authenticated.

Mycoplasma contamination

The cell lines were not tested for mycoplasma contamination.

Commonly misidentified lines
(See ICLAC register)

No commonly misidentified cell lines used.
